# Systemic capillary leak syndrome complicated by laryngeal edema after severe acute respiratory syndrome coronavirus 2 vaccination

**DOI:** 10.1002/ams2.700

**Published:** 2021-10-28

**Authors:** Maki Tanabe, Mayu Hikone, Kazuhiro Sugiyama, Yuichi Hamabe

**Affiliations:** ^1^ Tertiary Emergency Medical Center (Trauma and Critical Center) Tokyo Metropolitan Bokutoh Hospital Tokyo Japan; ^2^ Tokyo Emergency Room of Bokutoh Tokyo Metropolitan Bokutoh Hospital Tokyo Japan

## Disclosure

Approval of the research protocol: N/A.

Informed consent: The patient provided written consent for the publication.

Registry and registration no. of the study/trial: N/A.

Animal studies: N/A.

Conflict of interest: None.

Dear Editor,

Systemic capillary leak syndrome (SCLS) is a rare disease characterized by episodic exacerbation of hypovolemia, hemoconcentration, and hypoalbuminemia due to capillary hyperpermeability. Although the overall pathophysiology remains unknown, transient endothelial dysfunction is believed to be associated with the disease.^1^ Systemic capillary leak syndrome is diagnosed clinically, often requiring exclusion of other differential diagnoses such as sepsis, angioedema, and anaphylactic shock. Exacerbation of SCLS could be life‐threatening, leading to hypovolemic shock and multiple organ dysfunction as well as compartment syndrome and thromboembolic events as complications.^2^ Herein, we describe a patient who had an exacerbation of SCLS requiring intensive care after receiving a second dose of the severe acute respiratory syndrome coronavirus 2 (SARS‐CoV‐2) vaccine.

A 60‐year‐old woman presented to the emergency department with a 1‐day history of fever, dyspnea, and nausea. She received a series of SARS‐CoV‐2 vaccinations (BNT162b2; Pfizer‐BioNTech), two shots given 3 weeks apart, with the second dose given 2 days before the visit. Her medical history was relevant to a diagnosis of SCLS, with a life‐threatening attack of capillary leakage, complicated by cardiac arrest, that required intensive care. Her vital signs at presentation were: body temperature, 37.1°C; blood pressure, 81/60 mmHg; pulse rate, 122 beats/min; respiratory rate, 16 breaths/min; and oxygen saturation, 97% with ambient air. She was also alert, with oriented consciousness. Laboratory results showed an increased hematocrit level of 47% and a hemoglobin level of 15.1 g/dl. Nasal swab polymerase chain reaction testing for SARS‐CoV‐2 yielded negative results, and imaging studies did not indicate an infection source.

Although her hypotension and tachycardia temporarily resolved after a bolus of fluids, she continued to require a large amount of fluid, which made us consider SCLS possibly provoked by SARS‐CoV‐2 vaccination. She was admitted to the intensive care unit and required further resuscitative treatment with fluid and vasopressors. On hospital day 2, she developed hoarseness due to laryngeal edema and was intubated to secure her airway. During the first 24 hours, she received 12 L of fluids, but her hematocrit level and hypoalbuminemia worsened (Table 1). The increased fluid requirement gradually resolved after day 3, and she was extubated on day 6. She was discharged from the intensive care unit on day 10 and was discharged home on day 18.

**Table 1:** Laboratory results of a 60‐year‐old woman with systemic capillary leak syndrome complicated by severe acute respiratory syndrome coronavirus 2 vaccination

Variables	Reference range	At admission	12 h	Day 2	Day 3	Day 4	Day 5
White blood cells (/µl)	3300–8600	8500	27000	31600	28700	16900	11700
Hemoglobin (g/dl)	13.7–16.8	15.6	16.8	17.3	16.1	10.3	10.0
Hematocrit (%)	40.7‐50.1	47.0	51.4	53.4	50.2	31.5	30.0
Platelets (×10^4^/µl)	13.0–35.0	20.6	15.9	13.7	9.9	7.1	7.3
Albumin (g/dl)	4.1–5.1	3.6	1.7	1.8	1.5	1.9	2.2
Creatinine (mg/dl)	0.65–1.07	0.93	0.88	0.86	0.73	0.66	0.61
C‐reactive protein (mg/dl)	0.00–0.14	1.50	NA	2.74	5.88	4.69	2.83

Notes: NA, values with no data.

Adverse reactions to SARS‐CoV‐2 vaccination are usually local and mild; however, some serious reactions have been reported, including anaphylaxis, myocarditis, thromboembolic events, and Guillain‐Barré syndrome.^3^ Recent published reports describe acute exacerbation of SCLS 1–2 days after SARS‐CoV‐2 vaccination and suggest the possibility of SCLS as an adverse vaccine reaction.^4^, ^5^ Although we could not fully investigate all possible causes that triggered the SCLS, we were unable to identify any cause other than recent vaccination. Thus, we suspect that exacerbation of SCLS is related to vaccination.

Being a rare disease, SCLS may not be recognized or may be treated under a similar differential diagnosis. When patients present with hypovolemic shock from an unknown cause after SARS‐CoV‐2 vaccination, exacerbation of SCLS should be considered among the differential diagnoses.
